# Clinical Benefit of Antiviral Agents for Hepatocellular Carcinoma Patients With Low Preoperative HBV-DNA Loads Undergoing Curative Resection: A Meta-Analysis

**DOI:** 10.3389/fonc.2021.605648

**Published:** 2021-02-19

**Authors:** Kai-Xuan Liu, Jian-Guo Hong, Rui Wu, Zhao-Ru Dong, Ya-Fei Yang, Yu-Chuan Yan, Chun-Cheng Yang, Lun-Jie Yan, Sheng-Yu Yao, Hai-Chao Li, Xu-Ting Zhi, Tao Li

**Affiliations:** Department of General Surgery, Qilu Hospital, Shandong University, Jinan, China

**Keywords:** hepatocellular carcinoma, HBV reactivation, antiviral therapy, prognosis, HBV-DNA load

## Abstract

**Background and Aims:**

The clinical benefit of adjuvant antiviral therapy after curative therapy for HCC in patients with high preoperative HBV-DNA loads has been studied widely but that in patients with low preoperative HBV-DNA loads remains controversial. The purpose of this study was to determine the effect of antiviral treatment prophylaxis on HBV reactivation, overall survival (OS), and postoperative liver function in patients with low preoperative HBV-DNA levels undergoing curative resection.

**Methods:**

A meta-analysis was conducted by searching Web of Science, PubMed, Embase, and Cochrane Library until May 2020. We used REVMAN for data analysis and completed the study under the PRISMA guidelines.

**Results:**

Three randomized trials and seven cohort studies, comprising of 1,131 individuals, were included in the meta-analysis. Antiviral treatment significantly reduced the rate of HBV reactivation after curative treatment of HCC, with a pooled risk ratio of 0.12 (95% c.i. 0.07 to 0.21; P < 0.00001). The trials were consistently favorable for the antiviral group, with a pooled hazard ratio of 0.52 (95% c.i. 0.37 to 0.74; P = 0.0002) in respect of OS rate. However, by pooling the data from studies that reported ALT on the 30th day postoperatively, the result didn’t reach statistical significance (mean difference −4.38, 95% c.i. −13.83 to 5.07; P = 0.36). The I² values of the heterogeneity test for the above three comparisons are zero.

**Conclusion:**

Antiviral therapy during curative resection is effective in reducing HBV reactivation and improving OS rate in HCC patients with low viral load.

## Introduction

Hepatocellular carcinoma (HCC) is the sixth most frequent neoplasm worldwide and represents the third leading cause of cancer-related mortality (1). Surgical resection has been standard curative treatment for HCC patients with resectable tumors and unimpaired liver function (2). Unfortunately, tumor recurrence rate was very high after curative therapy with a 5-year recurrence rate of 70% (3). Currently, a major challenge encountered in HCC therapy is improving the prognosis of surgical patients. Hepatitis B virus (HBV) viral load is considered to be an important factor in predicting tumor recurrence (4), and high serum HBV-DNA levels seem to be associated with poor prognosis after the curative HCC resection (5). Liver resection can cause HBV reactivation in most HBV-related HCC patients, and HBV reactivation was related to tumor recurrence (6). However, although it has been extensively established that antiviral therapy can reduce the risk of tumor recurrence, antiviral therapy decreasing HBV reactivation has very rarely been studied.

In addition to these, previous studies have shown that antiviral therapy can reduce the risk of tumor recurrence in patients with high or low preoperative HBV-DNA loads (HBV-DNA < 2,000 IU/ml) (7, 8). However, some conflicting findings that antiviral therapy can improve outcomes in patients with low preoperative HBV-DNA loads have been reported. Huang et al., using a randomized controlled trial, suggested that antiviral group had better outcomes in the recurrence-free survival (RFS) and overall survival (OS) (8). Another study demonstrated that antiviral treatment showed a survival advantage for patients with low preoperative HBV-DNA loads (9). On the contrary, a cohort study observed antiviral treatment had no effects on improving postoperative RFS and OS (10).

Therefore, the purpose of this meta-analysis was to evaluate the effect of antiviral therapy on HBV reactivation and survival in patients with low preoperative HBV-DNA loads undergoing curative resection.

## Methods

### Databases and Searches

The literature was searched by computer without language constraints, using PubMed, Web of Science, Embase, and the Cochrane Library until May 2020. The predefined search policies were combinations of the Medical Subject Heading terms: “Carcinoma, Hepatocellular,” “antiviral agents,” “virus activation,” “Hepatectomy,” “nucleotide analog,” “adefovir,” “entecavir,” “HCC,” “liver cancer,” “hepatic cancer,” “liver resection,” “surgical resection,” “radical resection,” “curative resection,” “hepatic resection,” and free text words. Through this retrieval method, 453 articles were retrieved. A manual review of the reference list of relevant articles identified four additional studies.

### Study Selection

The criteria for inclusion were: (1) randomized controlled trials (RCT), prospective or retrospective cohort studies or case–control studies that enrolled HBV-related HCC patients who received curative resection as the initial treatment and their serum HBV DNA level was <2,000 IU/ml; (2) consisting of adjuvant antiviral treatment and a control arm of placebo or no treatment after liver resection; (3) no previous antiviral therapy before surgery; (4) adequate follow-up data on HBV reactivation, postoperative liver function, and OS of HCC patients.

### Data Extraction and Synthesis

Two authors (KL and JH) independently extracted the following data from the published reports: first author, country of origin, year of publication, study design, study population characteristics, number of patients AVT/non-AVT, type of antiviral agent used, cut-off value of low HBV DNA level, number of patients reactivation, hazard ratio (HR) with 95% confidence interval (CI) for OS and the value of ALT (alanine aminotransferase) at the 30th day postoperatively. In the included literature, some studies included people with both high and low viral loads, and this study only included people with low viral loads in the literature. Any disagreement between them was resolved by consensus. In this study, HBV reactivation after curative resection was considered as the primary endpoint. A consensus about the definition of HBV reactivation has been reached: it is an abrupt increase in serum HBV DNA levels by at least 1 log10 from baseline or its absolute value surpasses 10^9^ copies/Ml (11). OS and the value of ALT at the 30th day postoperatively served as secondary outcome. We used Risk of Bias in Non-randomized Studies of Interventions (ROBINS-I) to assess the methodological quality of non-randomized controlled studies. The risk of bias in each included RCTs was assessed by using Cochrane risk assessment tools.

### Statistical Analysis

The meta-analysis was done using the Cochrane Review Manager (RevMan, version 5.3; The Cochrane Collaboration, Copenhagen, Denmark) software. Statistical analysis for dichotomous variables time-to-event variables and continuous variables were respectively carried out using relative risk (RR), hazard ratio (HR), and mean difference (MD) as the summary statistic. A fixed effect model, the Mantel–Haenszel method, was used for homogeneous studies including dichotomous variables. Inverse variance method was used for pooling HR, MD. A value of P below 0.05 was considered statistically significant. The publication bias was evaluated by visual analysis of the funnel plots. We assessed heterogeneity by χ² and I² statistics. A fixed-effect model was used for comparison when heterogeneity was not substantial (I² < 25%).

## Results

### Characteristics of Studies Included in Meta-Analysis

**Figure 1:**
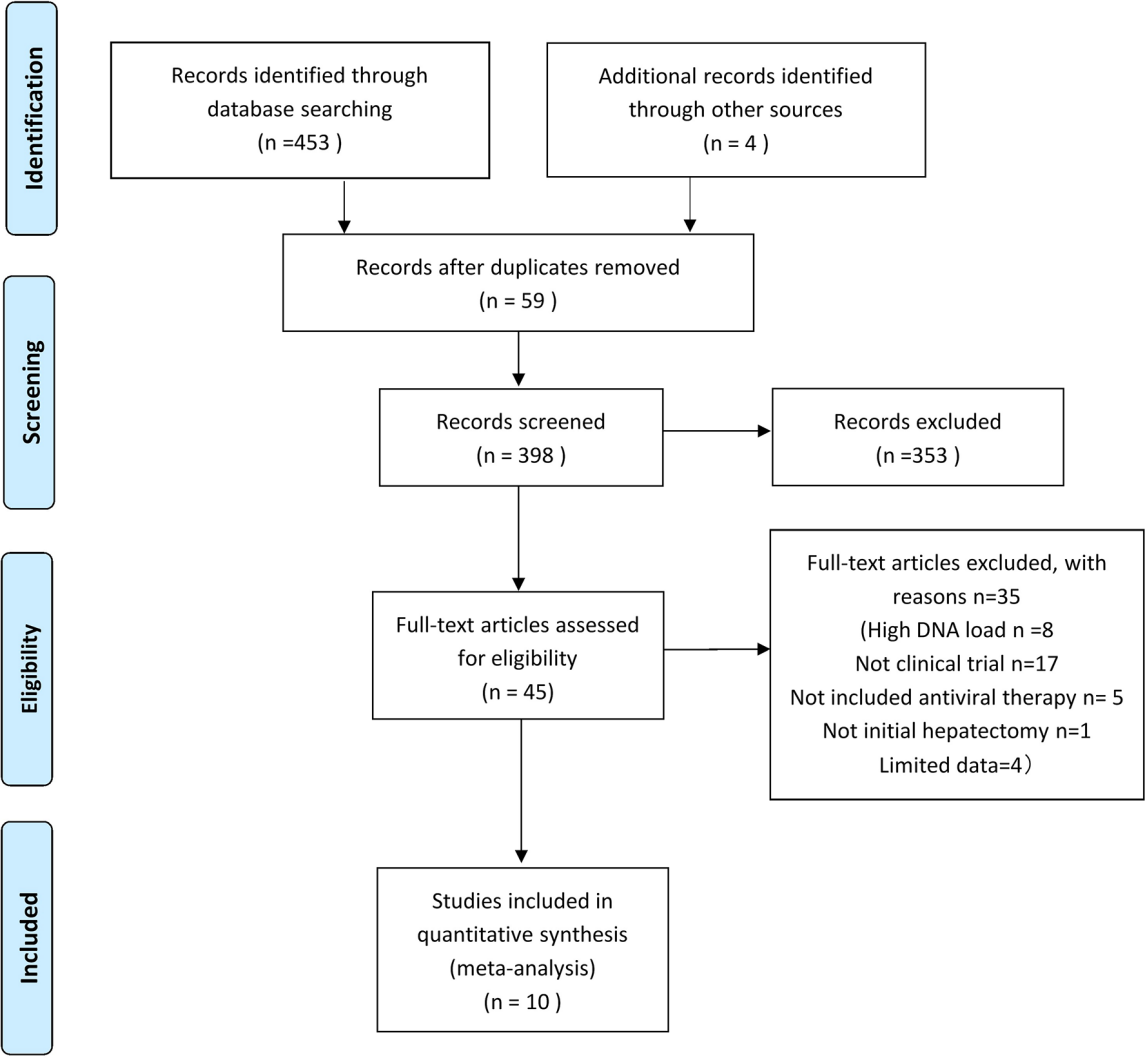
Study flow chart of the data extraction process and selection of studies for meta-analysis.

There were 453 articles identified through the systematic search and four additional manually searched articles, which comprised 449 HCC patients receiving antiviral therapy and 682 HCC patients without antiviral therapy (**Figure 1**). The main characteristics of the studies assessed by meta-analysis are outlined in **Table 1**. The main treatment in all the included studies was surgical resection. All studies were performed in Asia, among which nine were from China (8–10, 12–16, 19), and the other one was from Republic of Korea (17). Among the reviewed studies, three were RCTs and seven were NRCTs. All included articles were published between 2012 and 2019.

**Table 1:** Comparisons of the studies included for meta-analysis.

Study id	Country	No. of patients AVT/non-AVT	Study type	M/F	Type of antiviral agent used	Cut-off value of low HBV DNA (No.)	No. of reactivation	OS(HR,95%CI)	30days ALT
Chen et al. (12)	China mainland	20/54	prospective study	17/3 47/7	Entecavir	<500 IU/ml	1/20 15/54	N/A	N/A
Chen et al. (13)	China mainland	51/154	retrospective cohort study	48/3 131/23	Entecavir (93)* lamivudine(84) adefovir dipivoxil (13) telbivudine (2)	<2,000IU/ml	N/A	0.641 [0.215, 1.066]	N/A
Gong et al. (14)	China mainland	66/108	prospective study	58/8 92/16	entecavir	<500 IU/ml	2/66 30/108	N/A	35.6 ± 26.1 45.1 ± 11.8
Huang et al. (8)	China mainland	100/100	RCT	87/13 86/14	telbivudine	<2,000 IU/ml	3/100 25/100(1year)	0.549 [0.362, 0.832]	N/A
Huang et al. (15)	China mainland	27/34	prospectively study	N/A	N/A	<1.0 × 10^3^ copies/ml	1/27 4/34	N/A	N/A
Huang et al. (16)	China mainland	28/29	RCT	N/A	telbivudine	<1.0 × 10^4^ copies/ml	1/40 14/44	N/A	22 ± 8.9 24.8 ± 8.7
Lee et al. (17)	Republic of Korea	13/20	retrospective cohort study	N/A	nucleos(t)ide analog	Undetectable, <15 IU/ml	1/13 10/20	N/A	N/A
Liu et al. (18)	China mainland	63/102	RCT	43/20 67/35	entecavir	HBV-DNA-negative	2/63 13/102	N/A	35.3 ± 16.1 37.3 ± 16.2
Xu et al. (10)	China mainland	37/37	cohort study	32/5 31/6	entecavir	<100 IU/ml	2/35 3/34	0.375 [0.168, 0.834]	N/A
Yuan et al. (19)	China mainland	44/44	cohort study	32/12 38/6	entecavir	<8,866 copies/ml =1,773 IU/ml	1/44 11/44	N/A	31.8 ± 22.4 43.7 ± 19.2

Os, Overall survival; HR, Hazard Ratio; CI, Confidence Interval; ALT, Alanine aminotransferase; AVT, antiviral therapy; *The figures in brackets denote the number of patients.

**Figure 2:**
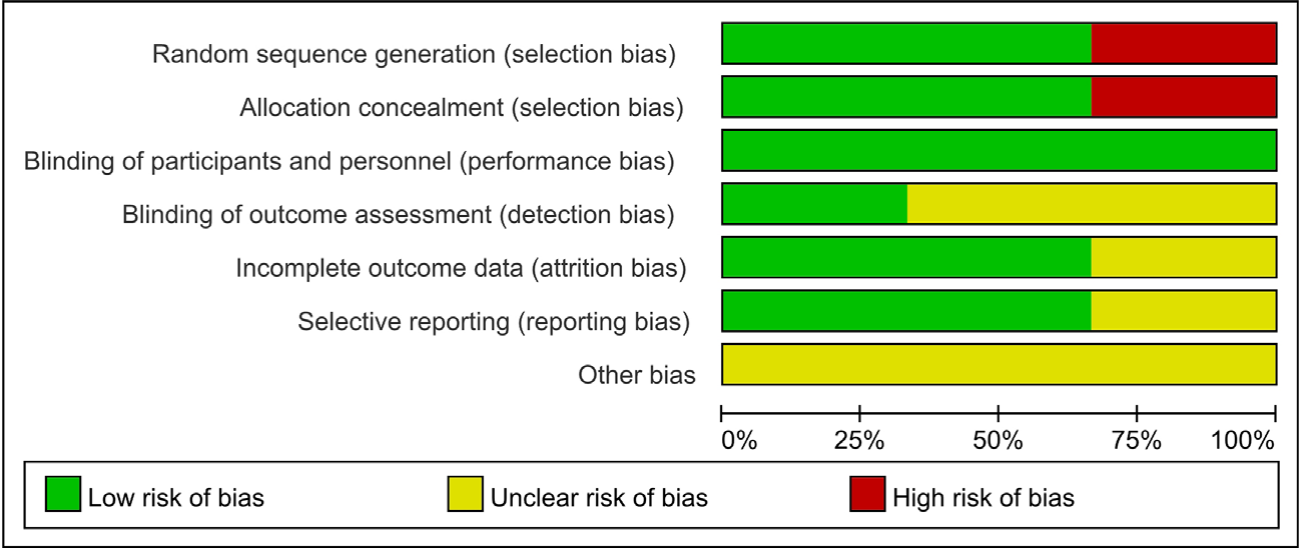
Risk of bias graph: review authors; judgements about each risk of bias item presented as percentages across all included RCT studies.

The methodological features of included non-randomized controlled studies are shown in **Table 2**. Among the seven observational studies included in the meta-analysis, only one was judged to have a serious risk of confounding bias. The remaining six studies were deemed to have a moderate (n = 4) or low (n = 2) risk of bias. For the three randomized trials identified among the included studies, we had some concerns regarding the risk of bias in the random sequence generation and allocation hiding due to the design flaw of study (**Figure 2**).

**Table 2:** Risk of bias assessment with ROBINS-I tool.

study id	Bias due to confounding	Bias in selection of participants in to the study	Bias in measurement of interventions	Bias due to departures from intended interventions	Bias due to missing data	Bias in measurement of outcomes	Bias in selection of the reported result	Overall Risk
Chen et al. (12)	Low	Low	Low	Low	Low	Low	Low	Low
Chen et al. (13)	Moderate	Moderate	Low	Low	Low	Low	Moderate	Moderate
Gong et al. (14)	Serious	Low	Low	Low	Low	Low	Low	Serious
Huang et al. (15)	Low	Moderate	Low	Moderate	Low	Low	Moderate	Moderate
Lee et al. (17)	Low	Low	Low	Low	Low	Low	Moderate	Moderate
Xu et al. (10)	Low	Low	Low	Low	Low	Low	Low	Low
Yuan et al. (17)	Low	Moderate	Low	Low	Low	Low	Low	Moderate

### Antiviral Therapy and HBV Reactivation

**Figure 3:**
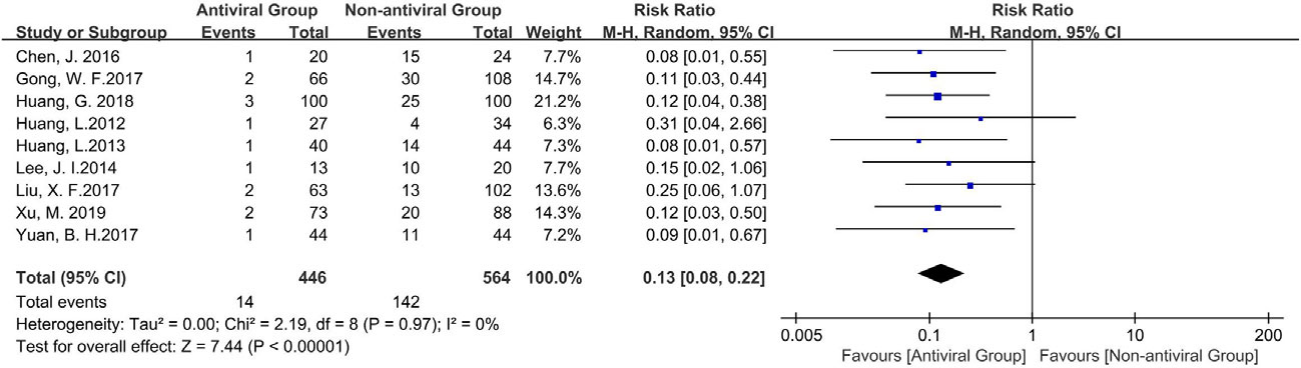
The Forest plot to compare the effect of anti-viral treatment vs. no treatment in HBV reactivation.

Nine of ten studies reported on HBV reactivation (**Figure 3**). Antiviral treatment significantly reduced the risk of HBV reactivation with a pooled risk ratio of 0.12 (95% c.i. 0.07 to 0.21); P < 0.00001) without statistical heterogeneity (I^2^ = 0%, P = 0.97 for χ2) in the meta-analysis.

### Antiviral Therapy and Overall Survival Rate

**Figure 4:**
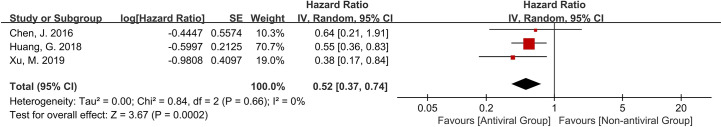
The Forest plot to compare the effect of anti-viral treatment *vs.* no treatment in overall survival rate.

Data were collected from three of the ten studies that reported 3-year OS. The trials were consistently favorable for the antiviral group, with a pooled hazard ratio of 0.52 (95% c.i. 0.37 to 0.74); P = 0.0002) without statistical heterogeneity (I^2^ = 0%, P = 0.66 for χ2) in the meta-analysis (**Figure 4**). Due to limited data available, the effect of antiviral therapy on local tumor recurrence or disease-specific survival could not be analyzed in this stud and no valid conclusions could be drawn.

### Antiviral Therapy and Liver Function

**Figure 5:**
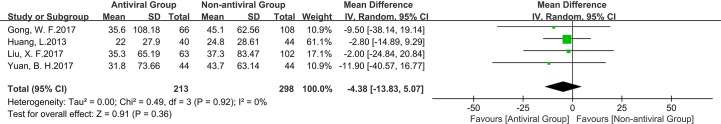
The Forest plot to compare the effect of anti-viral treatment *vs.* no treatment in liver function.

Though antiviral therapy often shows an advantage in long-term liver function of patients; however, due to limited data, our study did not conduct long-term liver function analysis. According to the analysis of the included literature, the available and meaningful data were ALT values at 30 days postoperatively. Pooled data from four of the ten studies revealed that ALT levels were lower, but not statistically significant (mean difference −4.38, 95% c.i. −13.83 to 5.07; P = 0.36), in patients receiving antiviral therapy. There was still no heterogeneity in this group (I^2^ = 0%, P = 0.92 for χ2) (**Figure 5**). The safety of antiviral therapy was well demonstrated, and none of the included studies provided data on adverse events associated with antiviral treatment.

### Publication Bias Analysis

**Figure 6:**
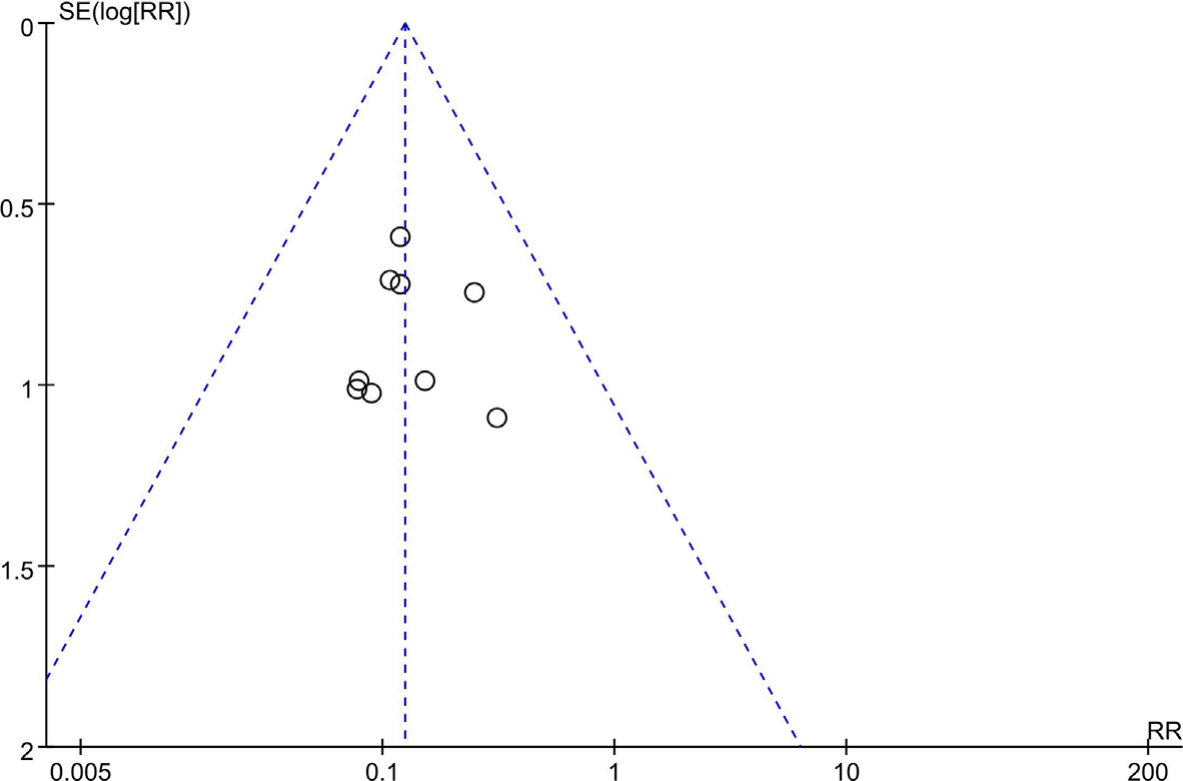
Funnel plot for the included trials—HBV reactivation. RR, risk ratio; SE, standard error.

No evidence of asymmetry was identified in funnel plots of OS and ALT. However, a funnel plot for HBV reactivation revealed a mild asymmetry, suggesting that there was publication bias (**Figure 6**).

## Discussion

It is an urgent problem for us to improve the prognosis of liver cancer patients. Previous studies have shown that for HCC patients with preoperative high HBV-DNA loads, antiviral therapy is of great significance for preventing recurrence and improving OS (20). However, there is still uncertainty as to whether there is benefit from antiviral therapy for HCC patients with low HBV DNA load. We conducted this meta-analysis to try to answer this question.

The mechanism of HBV reactivation remains unclear. Hepatic resection induced immunosuppression may increase the risk of HBV reactivation (21), which can impair liver function (4), aggravate liver cirrhosis (22), and increase the risk of HBV-related HCC recurrence (23). However, the main therapeutic mechanism of the antiviral drug was to prevent HBV replication and then reduce HBV reactivation regardless of HBV load (24, 25). Our results showed that antiviral treatment can effectively reduce HBV reactivation in HCC patients with low preoperative HBV-DNA loads undergoing curative resection. Tseng et al. revealed that HCC risk in hepatitis e antigen negative patients with low viral loads depends on HBsAg levels, not HBV DNA levels (26). Therefore, HBsAg seroclearance is considered as a safer antiviral treatment endpoint and has good off-treatment durability (27). We did not perform subgroup analysis because there was no significant heterogeneity.

Moreover, this study demonstrates that antiviral treatment is significant in improving OS rate in low viral load patients. To strengthen the study, we restricted the meta-analysis to 3-year OS rates. All included trials have available and comparable OS rates data at that time. Though antiviral treatment has no direct antitumorous effect, it can prevent HBV reactivation, inhibit hepatitis activity, reduce inflammation in liver remnants, and reverse cirrhosis and liver dysfunction (28). The hepatitis activity in the non-tumorous liver is known to be associated with tumor recurrence after hepatectomy. Antiviral therapy can reduce HCC risks by downregulating hepatic inflammation and related nuclear signaling pathways that lead to neoplastic transformation (8). In addition, antiviral therapy can also reduce the expression of the HBx protein to levels insufficient to promote HCC development and enhance tolerance to therapy against recurrence and leads to increased OS (29).

Previous studies have revealed that antiviral therapy can significantly improve liver function in patients with HBV-related HCC (28). In the present study, there was no statistical difference in ALT level on the 30th postoperative day between antiviral groups and non-antiviral groups. During the perioperative period, surgical factors such as hepatectomy induced severe damage of liver cells, the volume of liver parenchyma resected, and clamping of hepatic vessel may play a greater role in liver function recovery. Moreover, most of patients received antiviral therapy immediately when they developed HBV reactivation, which prevented the progress of hepatitis caused by replication of virus. In fact, antiviral therapy often shows an advantage in long-term liver function of patients, especially in patients with decompensated cirrhosis (30) and the improvements became most apparent after 9 months (31). Liu et al. also revealed that the treatment group had better postoperative liver function than the control group after a 1-year follow-up (18), which suggested antiviral treatment may have the advantage in long-term prognosis.

Certain limitations must be considered when interpreting study findings. First, although we conducted an extensive literature search, only three RCTs related to this theme were included in this meta-analysis, and one of the ten studies has serious risk of bias. There is concern with lacking well-designed prospective clinical trials in the literature. More well-designed RCTs with large sample sizes of patients are required in future analysis. Second, patients were treated with variable antiviral agents, including IFN and Nucleotide Analogue, and different timing of antiviral therapy and durations also have influence on the HBV reactivation. Third, all included studies were invariably conducted in Asia. The major risk factors for HCC vary from region to region. The high HCC rates in parts of Asia largely reflect the elevated prevalence of HBV infection. Seven genotypes (A to G) of HBV identified are associated with the disease progression and long-term outcome of HBV infection (32), and Genotypes B and C are prevalent in Asia (33). In most high risk HCC areas, such as China and South-East Asia, the key determinants are chronic HBV infection, and China alone accounting for about 50% of the total number of cases and deaths of HCC occurred worldwide (34). Therefore, it is inevitable that most HBV-related HCC studies are from Eastern countries especially from China (35). Additional studies are needed to better understand the effects of antiviral therapy on survival of HCC among other ethnic or geographic regions.

## Conclusions

In conclusion, the present analysis demonstrates antiviral therapy can effectively reduce HBV reactivation and improve OS rate in patients with low viral load. Therefore, we recommend antiviral therapy for patients with HBV-associated HCC after hepatectomy without delay, regardless of the viral load.

## Data Availability Statement

The original contributions presented in the study are included in the article/supplementary material. Further inquiries can be directed to the corresponding author.

## Author Contributions

Study design: K-XL, J-GH, RW, Z-RD, Y-FY, Y-CY, C-CY, L-JY, S-YY, H-CL, X-TZ, TL. Data collection: K-XL, J-GH. Data analysis: KX-L, J-GH. Writing: K-XL, TL. All authors contributed to the article and approved the submitted version.

## Funding

This study was supported by the Taishan Scholars Program for Young Experts of Shandong Province (tsqn20161064), the National Natural Science Foundation of China (Grant No. 81874178 and 82073200).

## Conflict of Interest

The authors declare that the research was conducted in the absence of any commercial or financial relationships that could be construed as a potential conflict of interest.
